# Coordinating Proliferation, Polarity, and Cell Fate in the *Drosophila* Female Germline

**DOI:** 10.3389/fcell.2020.00019

**Published:** 2020-02-04

**Authors:** Taylor D. Hinnant, Julie A. Merkle, Elizabeth T. Ables

**Affiliations:** ^1^Department of Biology, East Carolina University, Greenville, NC, United States; ^2^Department of Biology, University of Evansville, Evansville, IN, United States

**Keywords:** cell cycle, reproduction, stem cell, germ cell, ovary, oogenesis, gametogenesis

## Abstract

Gametes are highly specialized cell types produced by a complex differentiation process. Production of viable oocytes requires a series of precise and coordinated molecular events. Early in their development, germ cells are an interconnected group of mitotically dividing cells. Key regulatory events lead to the specification of mature oocytes and initiate a switch to the meiotic cell cycle program. Though the chromosomal events of meiosis have been extensively studied, it is unclear how other aspects of oocyte specification are temporally coordinated. The fruit fly, *Drosophila melanogaster*, has long been at the forefront as a model system for genetics and cell biology research. The adult *Drosophila* ovary continuously produces germ cells throughout the organism’s lifetime, and many of the cellular processes that occur to establish oocyte fate are conserved with mammalian gamete development. Here, we review recent discoveries from *Drosophila* that advance our understanding of how early germ cells balance mitotic exit with meiotic initiation. We discuss cell cycle control and establishment of cell polarity as major themes in oocyte specification. We also highlight a germline-specific organelle, the fusome, as integral to the coordination of cell division, cell polarity, and cell fate in ovarian germ cells. Finally, we discuss how the molecular controls of the cell cycle might be integrated with cell polarity and cell fate to maintain oocyte production.

## Introduction

Successful sexual reproduction requires high-quality haploid gametes. In many organisms, germ cells (which produce gametes) initially divide mitotically to form clusters of germ cells, from which differentiated oocytes or sperm arise. Because chromosome number remains constant across generations in sexually reproducing organisms, germ cells must switch to a meiotic cell cycle, wherein one round of DNA replication is followed by two rounds of cell division. In females, this process is made further complex by two additional processes: the selection of a single oocyte from a pool of precursor cells and the subsequent loading of maternally-derived transcripts and nutrients necessary post-fertilization for early embryonic development. Gametogenesis must, therefore, coordinate meiosis with the complex development of the specialized characteristics of the oocyte.

**FIGURE 1:**
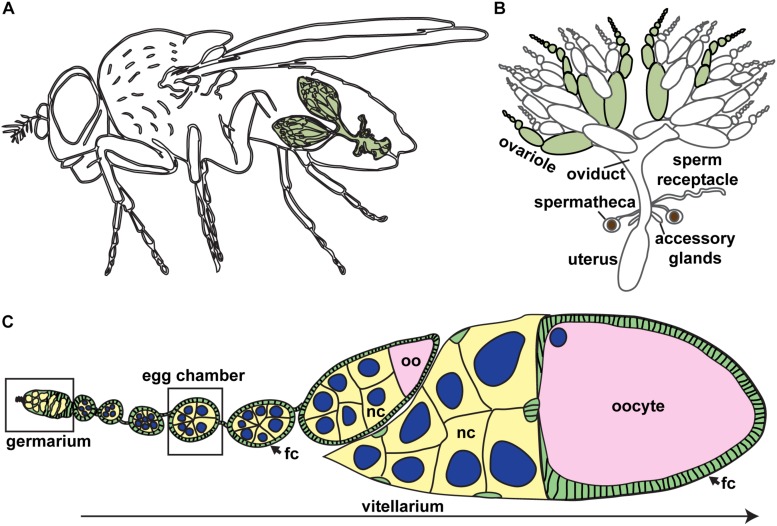
*Drosophila* ovaries are composed of linear arrays of developing oocytes. **(A)** Each female fruit fly has a pair of ovaries (green), each consisting of approximately 15–20 ovarioles. **(B)** The *Drosophila* female reproductive tract. Ovarioles are separated (green) to demonstrate ovariole structure. **(C)** Oogenesis begins in the germarium, where germ cells divide and are packaged into discrete units (egg chambers). Germ cells, yellow; oocyte, pink; somatic cells, green; nuclei of germ cells, blue. Most mature stages have been removed. fc, follicle cells; nc, nurse cells; oo, oocyte.

The fruit fly, *Drosophila melanogaster*, is a powerful experimental paradigm for oogenesis (Figures 1A,B). Oocytes arise from ovarian germline stem cells (GSCs), which self-renew and produce differentiating daughters called cystoblasts (Spradling, 1993; McLaughlin and Bratu, 2015). Cystoblasts mitotically divide exactly four times with incomplete cytokinesis, forming cysts of 16 interconnected germline progenitors. In each cyst, one cell differentiates to an oocyte fate and initiates meiosis, while the other 15 differentiate as nurse cells. Cysts are surrounded by and receive signals from somatic cells which promote germ cell divisions and shape oocyte morphology. The linear arrangement of oocyte production in the ovary allows for visualization of the proliferative capacity of GSCs and their daughters and the spatiotemporal regulation of meiosis (Figure 1C).

Over 100 years of elegant genetic and cytologic studies have clearly defined the chromosomal events that facilitate female meiosis and identified many of the genetic factors that regulate oocyte development. In particular, large scale genetic mutant screens provided critical insight into the molecular mechanisms that guide *Drosophila* oogenesis (Sandler et al., 1968; Baker and Carpenter, 1972; Schüpbach and Wieschaus, 1991; Sekelsky et al., 1999; Barbosa et al., 2007). Mutants were recovered based on easily scored phenotypes, such as egg production, egg morphology, and chromosome non-disjunction. For example, although mutants affecting oocyte determination were identified in genetic screens for maternal-effect lethal and female-sterile mutations, screen design did not permit recovery of homozygous lethal mutations (Schüpbach and Wieschaus, 1991). As a result, many genetic mutants that abrogate female fertility were described morphologically with respect either to cell biology (i.e., are oocytes made and if so, are they made correctly) or to meiotic recombination (i.e., did chromosomes exchange information correctly). More recently, screens employing powerful genetic tools to generate mutant cells specifically in the germline or ovarian soma increased our knowledge of the number of genes and genetic networks that underlie oogenesis (Morris et al., 2003; Denef et al., 2008; Ni et al., 2011; Horne-Badovinac et al., 2012; Czech et al., 2013; Jagut et al., 2013; Yan et al., 2014; Ables et al., 2016; Cho et al., 2018; Gao et al., 2019). These studies revealed that many fundamental molecular networks, particularly those that underlie asymmetric cell division during embryogenesis, are reiterated during the earliest steps of oogenesis to shape oocyte development.

In this review, we highlight the current knowledge of the early stages of oocyte production, particularly focusing on GSC proliferation and maintenance, cyst division, and oocyte specification, determination, and maintenance. Importantly, despite the progress in identifying critical molecular players, major questions regarding the mechanisms of early oogenesis remain unresolved. First, how is mitotic exit regulated in dividing cysts? While an intrinsic timing or counting mechanism seems likely, the molecular nature of this control has not been well-described. Second, how is the oocyte selected from a pool of 16 cells that share a common cytoplasm? Moreover, how is oocyte fate maintained once the cyst is surrounded by somatic follicle cells? These questions mirror larger, fundamental questions in the field regarding cell fate, cell cycle control, cell heterogeneity, and cell polarity, suggesting that future studies of the *Drosophila* germline will provide novel insights into how these mechanisms are orchestrated during development.

## The *Drosophila* Ovary: Development and Anatomy

### Germ Cell Establishment: Seeding Cells of the Future

**FIGURE 2:**
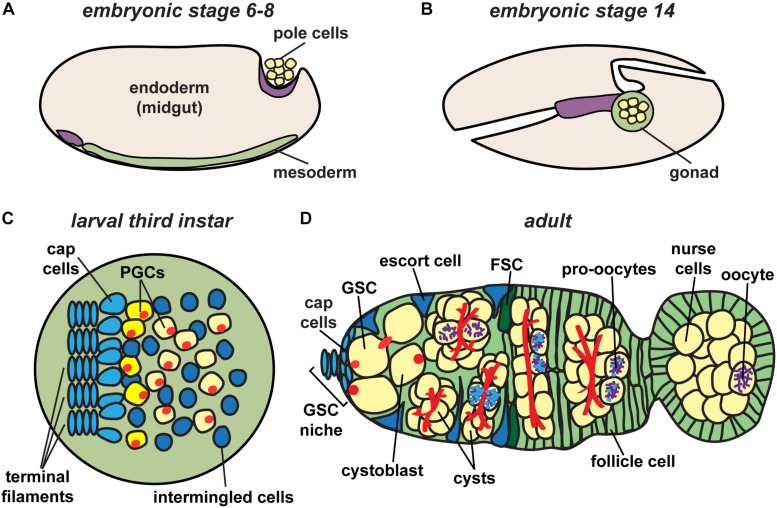
*Drosophila* ovary development. Germ cell precursors, known as pole cells, are specified early in development **(A)**, undergoing sexual specification after reaching the mesodermal gonad region **(B)**. Primordial germ cells (PGCs), which give rise to germline stem cells (GSCs), are specified in larval stages after receiving signals from surrounding somatic cells **(C)**. Following pupariation, GSCs in adult stages are positioned anterior to terminal filament and cap cells, and adjacent to escort cells **(D)**. Each germarium houses germline stem cells (GSCs) that give rise to each of the germline cell populations in the ovary, eventually resulting in the production of a mature oocyte. Germ cells, yellow; somatic cells, blue and green; fusomes, red; synaptonemal complex, purple.

Germ cell specification begins at the earliest stages of development when embryo polarity is first established. Among the first cellularization events in the *Drosophila* embryo are those of 10–15 posteriorly localized nuclei, specified to become primordial germ cells (also called pole cells) due to the presence of dense and abundant factors of the germ plasm in that region (Williamson and Lehmann, 1996). Upon cellularization, primordial germ cells undergo asynchronous divisions resulting in approximately 40 pole cells (Figure 2A). These cells then arrest in G_2_ phase of the cell cycle. Primordial germ cells (PGCs) begin their migration to the gonadal region in stage 10 of embryogenesis (approximately 5 h after egg laying) by invading the midgut epithelium (Figure 2B).

Formation of the ovary requires the coordinated development of primordial germ cells with somatic gonadal precursor cells. The latter includes three sub-populations of mesodermal cells that proliferate and migrate in concert with PGCs until they coalesce as an organ at the end of embryogenesis (Boyle and DiNardo, 1995; Broihier et al., 1998; Dansereau and Lasko, 2008). Somatic gonadal precursors differentiate into at least two populations of somatic cells that persist into adult stages: intermingled cells, which wrap PGCs; and terminal filament cells, which are the first somatic lineage to differentiate (Gilboa, 2015). During late larval stages, terminal filament cells in the anterior of the ovary extend into stacks, called terminal filaments (Figure 2C) (Godt and Laski, 1995; Sahut-Barnola et al., 1996; Lengil et al., 2015). Terminal filament cells recruit other somatic gonadal precursor cells to become cap cells, which adhere to the anterior end of the developing terminal filaments (Gilboa, 2015). PGCs that lie closest to the cap cells will be established as GSCs during pupariation (Asaoka and Lin, 2004). Remaining PGCs form the first “wave” of adult oogenesis, making up the first 3–6 oocytes produced (Figure 2D). Importantly, yolk uptake in *Drosophila* (called vitellogenesis) occurs exclusively during adult life (King, 1970). Thus, egg chambers formed by the non-stem cell PGCs do not complete development until after the female emerges from the pupal case. The number of larval/pupal terminal filament stacks thus predetermines the number of independent adult ovarioles separated by basement membrane and muscle, forming the egg-producing units of the adult ovary (Hodin and Riddiford, 2000; Sarikaya et al., 2012).

### Oogenesis in the Adult Female: A Dance in 14 Stages

Adult ovarioles resemble a developmental “assembly line” of oocytes (Figure 1C). As early as studies by Lubbock in the 1850s, the ovariole was considered a tube, which generates egg chambers at the apex and ovulates fully developed eggs to the oviduct at the base (reviewed in Telfer, 1975). Each ovariole consists of two morphologically distinct regions: the germarium, which lies at the anterior tip of each ovariole; and the vitellarium, consisting of increasingly larger egg chambers. In each egg chamber, a monolayer of somatic epithelial cells called follicle cells surrounds a single oocyte and 15 supporting nurse cells, collectively called a germline cyst (Figure 1C). Egg chamber development can be subdivided into 14 discrete, yet continuous stages; however, because the time necessary for the completion of each stage of oogenesis is variable, most ovarioles contain only seven or eight egg chambers at a given time (Spradling, 1993).

**FIGURE 3:**
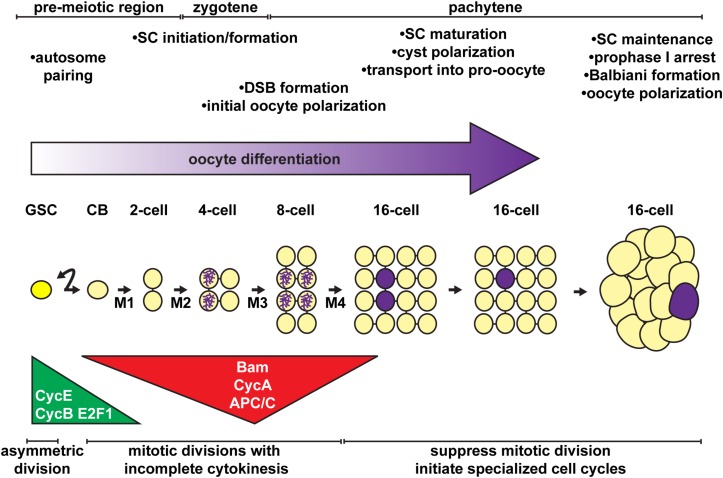
Stem cell activity, mitotic division, cell polarity, and the onset of meiosis must be coordinated for proper oocyte differentiation. Oocytes arise from the asymmetric divisions of the germline stem cell (GSC), which produce a differentiating daughter (cystoblast, CB). The cystoblast divides exactly four times (M1 – M4) with incomplete cytokinesis, forming a 16-cell cyst. Major regulatory mechanisms and meiotic events (timeline at top) are temporally coordinated with the mitotic divisions of the cystoblast/cysts (timeline at bottom). Germ cells, yellow; oocyte, purple.

In the anterior half of the germarium, cysts arise from the mitotic expansion of GSCs and their differentiating daughters (Figures 2D, 3). GSCs divide mitotically with asymmetric division, giving rise to two cells of unequal fates: a new stem cell and a cystoblast destined for differentiation (Schüpbach et al., 1978; Wieschaus and Szabad, 1979; Lin and Spradling, 1993; reviewed in: Xie, 2013; Gleason et al., 2018; Drummond-Barbosa, 2019; Kahney et al., 2019). The cystoblast undergoes exactly four rounds of mitotic division with incomplete cytokinesis, creating interconnected 2, 4, 8, and 16 cell cysts (King, 1970; Spradling, 1993). Cytoskeletal protein-rich ring canals, modified into stable intracellular bridges from the former cytokinesis contractile rings, maintain open connections between adjacent cyst cells, called cystocytes (Ong and Tan, 2010; Lu et al., 2017). Cyst division invariantly occurs in a stereotypical pattern, wherein the first two daughter cells (the M1 division products) develop four ring canals and are positioned in the center of the cyst (King, 1970; Spradling, 1993). One of these two cells (called the pro-oocytes) will become the future oocyte, while the remaining 15 cells differentiate as nurse cells. Although the oocyte and nurse cells spend most of the 14 stages as individual cells connected through cytoplasmic ring canals, they are ultimately joined into a single structure ready for fertilization. The process of nurse cell dumping streams contents of the nurse cell cytoplasm into the oocyte at Stage 10 (reviewed in Quinlan, 2016). At this stage, nurse cells enter programed cell death and gradually shrink in size through the remaining stages of oogenesis (reviewed in Peterson et al., 2015; Yalonetskaya et al., 2018).

Another critical process is the formation of the exterior features that provide environmental protection for both the oocyte and the developing embryo. The chorion and eggshell, the final outer layers of the oocyte, are deposited by specialized populations of somatic follicle cells, derived from a follicle stem cell population in the germarium (reviewed in McLaughlin and Bratu, 2015; Duhart et al., 2017; Osterfield et al., 2017). Follicle stem cells lie just posterior to the escort cells, and produce daughter cells that proliferate to fully encapsulate each cyst in a single monolayer (Figure 2D) (Margolis and Spradling, 1995; Nystul and Spradling, 2007; Reilein et al., 2017; reviewed in: Sahai-Hernandez et al., 2012). Centripetal migration of the follicle stem cell daughters (precursor follicle cells) also shapes the cyst, flattening it first into a lens-shaped structure with the two pro-oocytes at the center (King, 1970; Grieder et al., 2000). In the posterior-most region of the germarium, which also designates the first stage of oogenesis, cyst polarity is established and the cyst becomes round, thereby positioning the oocyte as the posterior-most cell of the cyst. Simultaneously, precursor follicle cells continue to migrate toward the center of the germarium, pinching the epithelium into a columnar layer of cells called a stalk and effectively separating individual egg chambers. Stalks continue to connect egg chambers through the remainder of oogenesis, even as cysts grow in size, rotate to shape the egg, and develop final structural features (reviewed in Cetera and Horne-Badovinac, 2015; Duhart et al., 2017).

### The Fusome Plays a Central Role in Early Oocyte Development

**FIGURE 4:**
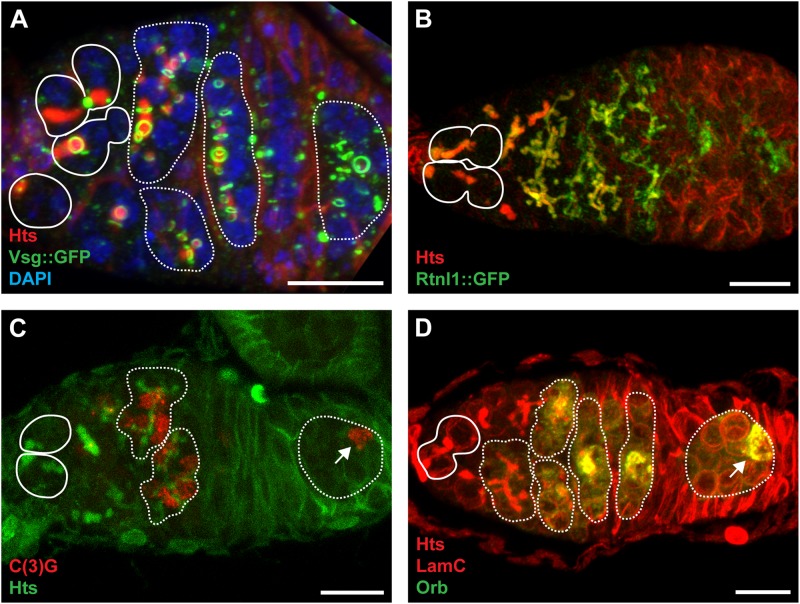
The fusome is composed of ER-like membranes and cytoskeletal proteins and is integral to cystocyte division and oocyte specification. **(A)** Confocal maximum intensity projection (5 μm z-depth) of a representative *vsg:GFP* germarium labeled with anti-Hts (red; fusomes and follicle cell membranes), anti-GFP (green; ring canals), and DAPI (blue; nuclei). Image modeled after Hinnant et al. (2017). **(B)** Confocal maximum intensity projection (33 μm z-depth) of a representative *Rtnl1:GFP* germarium labeled with anti-Hts (red; fusomes and follicle cell membranes) and anti-GFP (green; fusomes and endoplasmic reticulum). Image modeled after Röper (2007). **(C)** Confocal maximum intensity projection (5 μm z-depth) of a representative germarium labeled with anti-C(3)G (red; synaptonemal complexes) and anti-Hts (green; fusomes and follicle cell membranes). Image modeled after Page and Scott Hawley (2001) **(D)** Confocal maximum intensity projection (10 μm z-depth) of a representative germarium labeled with anti-Hts (red; fusomes and follicle cell membranes), anti-LamC (red; nuclear envelopes), and anti-Orb (green; presumptive oocytes). Image modeled after Tan et al. (2001). Circles demarcate germline stem cells; dotted lines demarcate cysts; arrows indicate oocytes. Scale bar, 10 μm.

**FIGURE 5:**
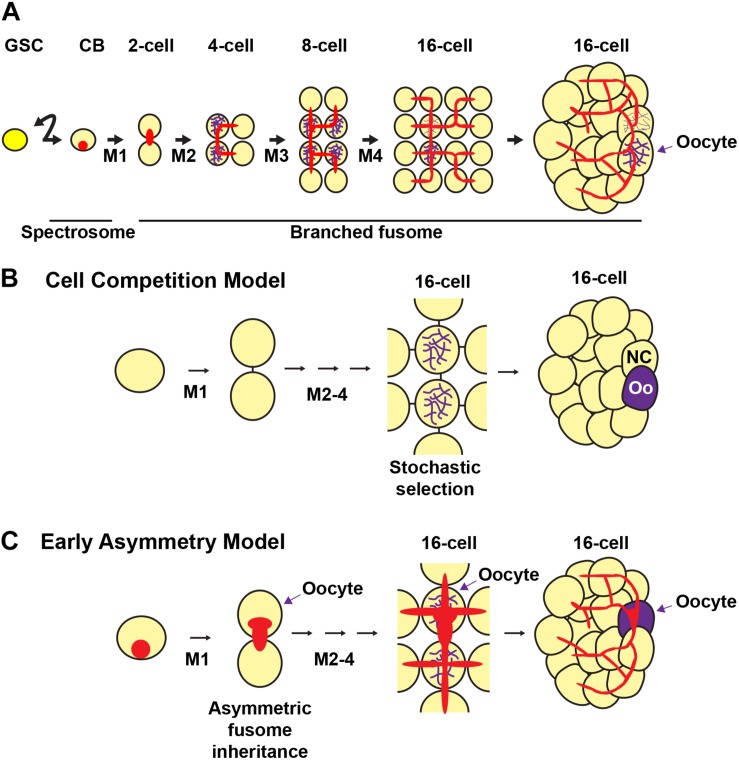
Fusome formation during cyst development and models of oocyte selection. The germline stem cell (GSC) divides asymmetrically to produce a cystoblast (CB). The cystoblast undergoes four mitoses (M1-4) with incomplete cytokinesis to form a 16-cell cyst. **(A)** The cystoblast contains a membranous structure called a spectrosome (red). During each mitotic division, the membranous material interconnects each of the cells of the cyst to form a branching fusome. The synaptonemal complex (purple) marks the pro-oocytes. **(B,C)** Models of oocyte selection. **(B)** Cell competition model. The pro-oocytes initiate meiosis I and are initially equally likely to become oocytes. Stochastic competition occurs between the pro-oocytes in the 16-cell cyst to result in a “winning” oocyte (Oo) and a “losing” nurse cell (NC). **(C)** Early asymmetry model. The fusome and its associated molecular factors are inherited asymmetrically from the first mitotic division, in which the original cystoblast retains more fusome material and is selected as the oocyte from this early stage. The fusome is associated with the Balbiani body, the mitotic spindle, and oocyte-specific mRNAs and proteins that may be critical for oocyte selection. Germ cells, yellow; oocytes, purple; fusomes, red.

A distinguishing feature of *Drosophila* PGCs, GSCs, and early germ cells is a large, dynamic cytoplasmic structure which extends through ring canals to all of the connected cystocytes. Telfer coined the term “fusome” to describe a cylindrical gel extending through the ring canals, notably devoid of ribosomes and mitochondria in transmission electron micrographs (King, 1970; Telfer, 1975). During GSC division, a small plug of fusome material is deposited in the differentiating daughter cell (the prospective cystoblast; also termed the pre-cystoblast) (Figures 2D, 4A) (de Cuevas and Spradling, 1998; Huynh, 2006; Ong and Tan, 2010; Ables and Drummond-Barbosa, 2013). Fusome material accumulates concomitant with disassembly of the mitotic spindle at each mitotic division of the cystoblast, forming a continuous branched structure (Figures 4B, 5A) (Koch and King, 1966; Mahowald, 1971; Lin and Spradling, 1995; de Cuevas and Spradling, 1998). Fusome architecture (i.e., shape, size, and branching pattern) is, therefore, a defining morphological feature of cysts as they differentiate. Importantly, the fusome is critical for vesicular transport and likely enables communication between cyst cells as they progress through the cell cycle (McKearin, 1997; Huynh, 2006; Lu et al., 2017). For example, individual cyst cells are synchronized by the fusome to enter mitosis concurrently (Lin et al., 1994; de Cuevas et al., 1996). The fusome also anchors cystocyte centrosomes and mitotic spindles, establishing cell polarity and dictating the orientation of the mitotic division plane (Lin et al., 1994; Lin and Spradling, 1995; de Cuevas et al., 1996; Deng and Lin, 1997; Grieder et al., 2000). Indeed, Telfer noted that “cytoplasmic residues” of mitotic spindles appeared to join in the fusome (Telfer, 1975). Genetic mutants lacking fusomes fail to enter mitosis together, eventually blocking cyst division (Yue and Spradling, 1992; Lin et al., 1994; de Cuevas et al., 1996).

Extensive molecular studies demonstrated that the fusome is composed of both cytoskeletal proteins and membranous tubules (Figure 4B). Membrane cytoskeletal proteins, including the Adducin-like Hu-li tai shao (encoded by *hts*), Ankyrin, and alpha- and beta-Spectrins appear to form the core of the fusome structure, as mutations in *hts* abolish both cytoskeletal and membrane components (Lin et al., 1994; McKearin and Ohlstein, 1995; de Cuevas et al., 1996; Röper and Brown, 2004; Röper, 2007). Moreover, although vesicle- and cytoskeleton-associated proteins, including F-actin, the actin-capping protein Tropomodulin (encoded by *tmod*), and the microtubule-associated Par-1, localize to the fusome (Röper and Brown, 2004; Snapp et al., 2004; Röper, 2007; Lighthouse et al., 2008; Mathieu et al., 2013), the structure does not contain extensive microtubules (Warn et al., 1985; Theurkauf et al., 1993). Instead, membrane vesicles resemble those of the endoplasmic reticulum (ER) or Golgi apparatus (Snapp et al., 2004; Röper, 2007; Lighthouse et al., 2008). Proteins associated with the ER, including channel protein Sec61α, lumenal stress-associated Protein Disulfide Isomerase (PDI), and membrane proteins Reticulon-like 1 (Rtnl1) and TER94 accumulate as fusomes grow and branch (Figure 4B). To date, however, the only vesicle-associated protein demonstrated as essential for germ cell divisions and fusome integrity is the endosomal trafficking protein Rab11 (Bogard et al., 2007; Lighthouse et al., 2008).

### Models of Germ Cell Differentiation

Superimposed on the structural development of the oocyte as an individual package, the oocyte and nurse cells undergo their own unique differentiation programs. In the case of the oocyte, the initial stages of meiosis occur concurrently with the final two mitotic cyst divisions (reviewed in Rubin et al., 2016; Hughes et al., 2018). In dividing cysts (particularly following divisions M2 and M3; Figure 3), pro-oocytes can be experimentally distinguished from the nurse cells using several markers, including the presence of the meiotic synaptonemal complex (SC) (Figure 4C), cytoskeletal and nuclear morphology, oocyte positioning within the cyst, and accumulation of oocyte-specific factors (Figure 4D) (reviewed in Huynh and St Johnston, 2004; McKim et al., 2009). By the time egg chambers exit the germarium, most cyst cells will reverse their recombination efforts and exit meiosis, leaving only the true oocyte to remain in meiosis (reviewed in Hughes et al., 2018). The remaining 15 nurse cells enter the endoreplication cycle, replicating their genome without cell division, and synthesize proteins and mRNAs to be transported to the developing oocyte (reviewed in Lee et al., 2009; Shu et al., 2018). Initiation of endocycling in nurse cells, considered a hallmark of nurse cell differentiation, occurs only after a significant gap in developmental time, during which it is likely that the final stages of differentiation away from the oocyte program are completed. During this gap, the decision to become an oocyte or a nurse cell appears to retain some plasticity, as many genetic mutants (particularly in genes encoding oocyte polarity factors) revert from an oocyte fate to a nurse cell identity (Mach and Lehmann, 1997; Cox et al., 2001; Huynh et al., 2001a).

Two prevailing models posit how oocytes are selected during cyst development (Figure 5) (Spradling, 1993; Carpenter, 1994; McKearin, 1997; Huynh, 2006). The first model (Figure 5B) suggests that oocyte specification occurs after cystocyte mitoses are complete. This view emerged based on the observation that pro-oocytes (the two cystocytes with four ring canals), are initially indistinguishable (Mahowald and Strassheim, 1970). In region 2a of the germarium, the pro-oocytes and the two cystocytes with three ring canals all enter meiosis and form synaptonemal complexes between homologous chromosomes in meiotic prophase I (Carpenter, 1975; Carpenter, 1994). Symmetry is broken when the cyst progresses midway through the germarium, cyst division is complete, and the synaptonemal complex is restricted to the two pro-oocytes (González-Reyes et al., 1997; Huynh and Johnston, 2000; Page and Scott Hawley, 2001; McKim et al., 2002). The model predicts that stochastic competition between the two pro-oocytes results in random selection of the future oocyte by the accumulation of specific mRNAs, proteins, and/or organelles. The selected oocyte then remains in meiotic prophase I until stage 13 of oogenesis when it enters the first meiotic metaphase. For the determined oocyte to remain meiotically dormant, it condenses its chromatin to form the karyosome, a structure conserved among diverse organisms. Meanwhile, the other pro-oocyte exits meiosis and reverts to the nurse cell fate (King, 1970). The 15 nurse cells enter the endoreplication cycle, replicating their genome without cell division, and synthesize proteins and mRNAs that are transported to the developing oocyte (reviewed in Shu et al., 2018).

In contrast, the second model of oocyte specification suggests that the oocyte is specified at the time of the first mitotic division of the cystoblast, establishing an asymmetry that is maintained in subsequent mitoses (Figure 5C) (Yue and Spradling, 1992; Lin et al., 1994; Theurkauf, 1994a; de Cuevas and Spradling, 1998). Support for this model arises from studies showing that the fusome is asymmetrically inherited at every cyst division (Storto and King, 1989; Lin and Spradling, 1995; McGrail and Hays, 1997; de Cuevas and Spradling, 1998). This begins in the GSC, where one of the daughter cells (the presumptive cystoblast) inherits approximately two-thirds of the fusome (de Cuevas and Spradling, 1998). The fusome then continues to be asymmetrically distributed in the cystoblast mitotic divisions, resulting in one of the pro-oocytes having a larger piece of the fusome than any of the other cystocytes. The cell with the most fusome accumulates the oocyte-specific mRNAs *oskar* (*osk*) and *oo18 RNA-binding protein* (*orb*) (which are important for axis specification), receives the centrosomes of the other 15 cells, and is eventually specified as the oocyte (Grieder et al., 2000; Bolívar et al., 2001; Cox and Spradling, 2003). The fusome begins to break down by an unknown mechanism in post-mitotic 16-cell cysts, almost completely disappearing by the time the cyst buds off the germarium (de Cuevas and Spradling, 1998). Mutations in key fusome components, including *hts* and α*-spectrin*, disrupt oocyte specification, thereby suggesting that the fusome is necessary for proper oocyte specification (Yue and Spradling, 1992; Theurkauf et al., 1993; de Cuevas et al., 1996).

Since the proposition of these models, considerable genetic and molecular evidence suggests that selection and maintenance of the oocyte is a continuum, mediated by an internal timing mechanism in dividing cystoblasts/cysts and the polarizing activities of the fusome. It is worth noting that the language used to refer to oocyte fate establishment is often used inconsistently in the literature. We speculate that this is due to a general lack of understanding of the precise molecular events that lead the oocyte to its final fate during gametogenesis. Great progress in the field over the last 15 years has steadily increased the number of known molecular players, leading to a better fundamental grasp of oocyte development. In the remainder of this review, we, therefore, adopted the terms used in the field of developmental biology to describe the molecular steps leading to oocyte specification. Here, cell “specification” refers to an initial decision-making event in which the cell is diverted to a specific fate but remains competent to revert to an earlier step in the lineage. “Determination” refers to a stage at which this reversion in cell identity is no longer possible, although the cell can still become different fates down that lineage. Finally, “differentiation” is used to describe the entire process of cell fate, including the final functional state within the tissue and organism. By these definitions, the oocyte could be considered fully differentiated at egg activation when meiosis is completed. Although many molecular details surrounding these events have been well-described, the processes of oocyte specification and determination remain elusive and will be explored further herein.

## Activity of GSCs and Cystoblasts Establish a Pool of Undifferentiated Cells From Which Oocytes Are Specified

In *Drosophila*, oogenesis is maintained in adult females by the proliferative activity of GSCs. Loss of GSCs, either by experimental ablation, genetic mutation, decreased nutrition, or female aging, blocks oocyte production (reviewed in Drummond-Barbosa, 2019; Kahney et al., 2019). GSCs must maintain an undifferentiated state while also continuing to proliferate. Failure of the GSCs to enter the cell cycle or to suppress differentiation leads to stem cell loss and cessation of oocyte production. Alternatively, unregulated stem cell divisions or a block in differentiation lead to excess stem-like cells at the expense of differentiated cells, ultimately blocking oocyte production. The close association of somatic cells with germ cells established during embryogenesis is conserved in adults and dictates the balance between GSC self-renewal and cystocyte differentiation.

### Paracrine Signaling Promotes GSC Self-Renewal and Daughter Cell Differentiation

**FIGURE 6:**
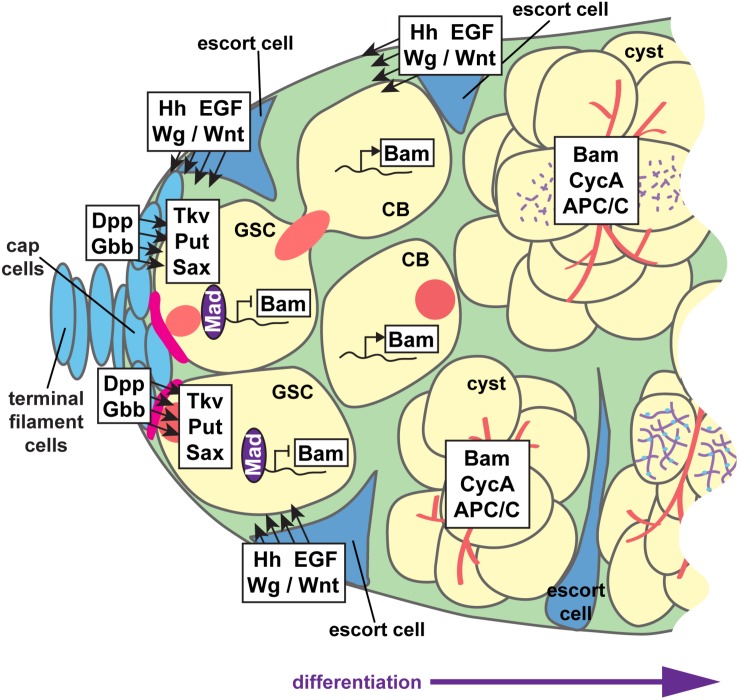
Paracrine signaling supports GSC self-renewal and cystoblast differentiation. Interwoven signaling pathways support germline stem cell (GSC) self-renewal and differentiation of the cystoblast (CB) and cystocytes to nurse cell or oocyte fates. GSCs are anchored to cap cells via adherens junctions (pink). Cap cells secrete the bone morphogenetic protein (BMP) ligands Dpp and Gbb, which are received by BMP receptors Tkv, Put, and Sax on GSCs. Activation of BMP signaling promotes Mad phosphorylation and transcription factor activity, which suppress transcription of the differentiation factor *bam*. The range of BMP ligands is limited by Hh, Wg/Wnt, and EGF ligands secreted from adjacent escort cells. Thus, Bam is produced in germ cells that do not receive sufficient BMP signals. Hh, Wg/Wnt, and EGF ligands also promote germ cell differentiation, at least in part by maintaining the long, thin, axonal-like escort cell projections that dynamically wrap cysts as they move posteriorly. Germ cells, yellow; somatic cells, blue; presumptive synaptonemal complexes, purple; fusomes, red.

In the anterior germarium, somatic cells form two paracrine signaling centers that collectively promote GSC self-renewal and germ cell differentiation (Figure 6). The anterior “stem cell niche” is formed by terminal filament cells and cap cells at the tip of each germarium (Figure 2D) (Xie, 2013; Drummond-Barbosa, 2019). GSCs are physically anchored to cap cells via adherens junctions and gap junctions, which are required for maintaining GSCs in the niche (Song et al., 2002; Gilboa et al., 2003). Cap cells secrete the bone morphogenetic protein (BMP) ligands Decapentaplegic (Dpp) and Glass bottom boat (Gbb) (Xie and Spradling, 1998; Song et al., 2004). BMP ligands are received through transducing receptors Punt (Put), Thickveins (Tkv), and Saxophone (Sax) on the cell periphery of GSCs (Xie and Spradling, 1998; Casanueva and Ferguson, 2004). GSCs extend short filopodia around the cap cells, sequestering Dpp and Gbb to promote GSC self-renewal and block differentiation (Wilcockson and Ashe, 2019). In GSCs, activated BMP receptors phosphorylate the transcription factor Mad, allowing it to translocate to the nucleus (Xie and Spradling, 1998; Kai and Spradling, 2003). The best-known target of Mad transcription in GSCs is the differentiation factor encoded by *bag-of-marbles* (*bam*). Dpp signaling through Mad represses *bam* transcription, suppressing differentiation in GSCs (Chen and McKearin, 2003; Song et al., 2004). *bam* encodes a ubiquitin-associated protein essential for cystoblast differentiation and mitotic division (McKearin and Spradling, 1990; Ohlstein and McKearin, 1997; Ji et al., 2017). Other factors, including the PIWI family proteins Piwi and Aubergine, similarly function to maintain GSC self-renewal by repressing *bam* (Ma et al., 2014, 2017; Rojas-Ríos et al., 2017); reviewed in Rojas-Rıós and Simonelig (2018). Bam promotes specific protein-protein interactions that bolster cystoblast differentiation (Li et al., 2013; Pan et al., 2014). Tight regulation of Dpp signaling and Bam activity thus constrains the GSC fate to cells closest to cap cells, allowing differentiation of more proximal cells toward the cystoblast fate.

A second signaling center, sometimes termed a “differentiation niche,” is formed by the somatic escort cells (also called inner germarial sheath cells) (Figure 2D). Escort cells promote germ cell differentiation and guide the posterior movement of differentiated cysts (Decotto and Spradling, 2005; Morris and Spradling, 2011; Banisch et al., 2017). Escort cells line the outside of the anterior germarium (regions 1–2) and send long, thin cellular protrusions into the center of the germarium, around the dividing germ cells (Kirilly et al., 2011; Morris and Spradling, 2011; Banisch et al., 2017). Escort cell protrusions dynamically wrap the dividing cystoblasts and are essential for mitotic division of the cysts, as well as the association of follicle cells with the germline at encapsulation. Like cap cells, escort cells also produce and secrete signaling molecules; Hedgehog, Wnt/Wg, Epidermal Growth Factor (EGF), insulin, and ecdysone signaling all function non-autonomously in escort cells to control germ cell differentiation (Morris and Spradling, 2012; Xuan et al., 2013; Eliazer et al., 2014; König and Shcherbata, 2015; Liu et al., 2015; Lu et al., 2015; Luo et al., 2015; Mottier-Pavie et al., 2016; Huang et al., 2017; Su et al., 2018; Wang and Page-McCaw, 2018; Mao et al., 2019). In general, many of these signals act to sustain the cytoskeletal structure and/or dynamics of escort cell protrusions. It has also been postulated that most escort cell signals limit the range of BMP signals emanating from cap cells, which blocks the undifferentiated (stem cell) state and promotes germ cell differentiation and mitotic division (Gao et al., 2019).

### Mitotic Divisions in GSCs

Not surprisingly, cell cycle regulation is essential for GSC proliferation and self-renewal (Figure 3). GSCs appear to progress through a G1/S/G2/M cell cycle, albeit with very short G1 and M phases and a very long G2 phase (Hsu et al., 2008; Ables and Drummond-Barbosa, 2013; Kao et al., 2015). In healthy young females, GSCs divide approximately every 12–14 h (Morris and Spradling, 2011). Protein levels of many cell cycle regulatory proteins, such as the G1- and S-phase proteins Cyclin E (CycE) and E2f1, are maintained nearly constantly across all phases of the cell cycle (Hsu et al., 2008; Ables and Drummond-Barbosa, 2013; Hinnant et al., 2017). GSCs require the activity of Cyclins and Cyclin-dependent kinases (Cdks) to progress through the cell cycle (Wang and Lin, 2005; Ables and Drummond-Barbosa, 2013; Chen et al., 2018). For example, loss of either the M-phase regulator Cyclin B (CycB)/Cdk1 or the S-phase regulator Cyclin E (CycE)/Cdk2 blocks GSC proliferation (arresting cells in G2 or G1, respectively). GSCs carrying mutations in the Cdk1 inhibitor *myt1* divide at faster rates (Jin et al., 2005; Kao et al., 2015), while GSCs defective for the essential S-phase transcription factor *E2f1* divide at slower rates (Jin et al., 2008; Hinnant et al., 2017). Loss of CycB cannot be rescued by overexpression of the G2/M regulator CycA, suggesting that CycB is specifically required for M-phase activities (Wang and Lin, 2005). Accordingly, Cyclin B is also required for abscission of the newly-divided cystoblast from the GSC in late mitosis, under the temporal control of Aurora B kinase (Mathieu et al., 2013).

While cell cycle control is intimately associated with maintaining an undifferentiated cell fate in GSCs, it remains largely unclear how this connection is achieved at the molecular level. Experimental analyses of protein null *CycA*, *CycB*, *CycE*, or *Cdk2* mutant GSCs have not been useful in this regard, as these cells are rapidly eliminated from the stem cell niche by unknown molecular mechanisms (Wang and Lin, 2005; Ables and Drummond-Barbosa, 2013). In contrast, *CycE* mutants with lower kinase activity can enter S-phase at a rate equivalent to wild-type GSCs, but fail to maintain the GSC fate (Ables and Drummond-Barbosa, 2013). Similarly, reduction of *CycA* mRNA in GSCs and their dividing daughters supports cell division and GSC self-renewal (Ji et al., 2017), albeit at reduced levels than wild-type GSCs. This suggests that cell fate and progression through the cell cycle are, at some level, molecularly distinct events. One possibility is that CycE/Cdk2 (and perhaps also CycA/Cdk1) substrates necessary for stem cell fate require a lower threshold of phosphorylation than do those that promote cell cycle timing. This could be accomplished by modulating the total levels of kinase activity (i.e., by Cdk1 and Cdk2) or by differential phosphorylation at multiple sites, as proposed in yeast models (Swaffer et al., 2016; Örd et al., 2019). Indeed, degradation of CycA (but not E2f1, CycB, or CycE) is essential to maintain GSC self-renewal, suggesting there is a window of the cell cycle at which total Cyclin/Cdk levels peak (Chen et al., 2009; Hinnant et al., 2017). A second possibility is that CycE/Cdk2 phosphorylates a key substrate necessary for cell fate, but independent of the canonical G1/S-phase regulators. Evidence for this hypothesis comes from recent studies in mammalian embryonic stem cells, where G1-phase cyclins directly phosphorylate the BMP effectors Smad2 and Smad3, inhibiting their translocation to the nucleus and blocking differentiation (reviewed in Liu et al., 2019). While a BMP signaling effector would be an attractive candidate substrate, current experimental tools have not uncovered an obvious correlation between BMP activation and cell cycle phase in GSCs (Ables and Drummond-Barbosa, 2013). Future studies exploring the role of the Retinoblastoma homolog Rbf will address both potential models and help identify key molecular targets that connect the GSC fate with cell cycle control. Rbf is a substrate of CycE that negatively regulates E2f1 and has been recently demonstrated to promote a differentiation-biased transcriptional program independently of its role in cell cycle control (Zappia et al., 2019).

Another key aspect of mitotic divisions in GSCs is that each division is asymmetric, giving rise to two daughters of unequal fates. Asymmetry is, in part, dictated by GSC adhesion on one pole to cap cells and subsequent activation of the small GTPase Rac at the GSC-cap cell interface, adjacent to the fusome (Deng and Lin, 1997; Song et al., 2002; Lu et al., 2012). Rac activity promotes localization of a microtubule-organizing centrosome at the GSC-cap cell interface and dictates the plane of GSC division (Lu et al., 2012). This enables asymmetric partitioning of the daughter centrosome and midbody to the parent GSC (Salzmann et al., 2014; Matias et al., 2015), but may also promote asymmetric localization of other factors critical for cell fate. Indeed, additional intrinsic factors have also been identified that molecularly distinguish the parent GSC from the presumptive cystoblast. GSCs have high levels of RNA polymerase I-dependent transcription, necessary for proliferation and self-renewal (Zhang et al., 2014). Wicked, a conserved nucleolar protein required for rRNA maturation, is also enriched in GSCs (Fichelson et al., 2009). Live imaging studies demonstrated that Wicked is concentrated in cytoplasmic particles that asymmetrically segregate to the parent GSC during mitosis. While rRNA transcription regulates GSC self-renewal by promoting the expression of the BMP effector Mad, rRNA transcription and maturation likely also act with other BMP-independent mechanisms to promote asymmetric division (Fichelson et al., 2009; Zhang et al., 2014).

Fluorescence-based reporters of G1/S and G2/M phases demonstrated that cell cycle timing in GSCs and cystoblasts is largely similar (Hinnant et al., 2017). This is likely because the GSC and the newly-formed cystoblast remain physically connected by a thin cytoplasmic bridge after mitosis until completion of S-phase of the subsequent cell cycle (de Cuevas and Spradling, 1998; Ables and Drummond-Barbosa, 2013; Mathieu et al., 2013). Complete abscission of the cystoblast is timed by the opposing activities of CycB/Cdk1 and the chromosomal passenger complex, composed of Aurora B kinase and Survivin (Mathieu et al., 2013). Membrane cleavage at abscission requires the Endosomal Sorting Complex Required for Transport-III (ESCRT-III) complex and the scaffold protein ALIX, and is negatively regulated by Aurora B (Eikenes et al., 2015; Matias et al., 2015). Following abscission, a brief pulse of transcriptional silencing in the presumptive cystoblast prior to Bam expression is induced by the transcriptional repressor Polar granule component (Flora et al., 2018). This allows for the accumulation of CycB, promoting cell cycle resumption and differentiation in the cystoblast. Intriguingly, CycB, Survivin, and the ESCRT-III complex protein Shrub are enriched in the fusome of GSCs and cystoblasts, underscoring the importance of the fusome for coordinating cell division and polarity (Mathieu et al., 2013; Matias et al., 2015). One possibility is that the fusome acts as a conduit to physically concentrate cell cycle regulators in a specific subcellular domain, imparting temporal and spatial control over asymmetric division and abscission. It is also tempting to speculate that the initial fusome asymmetry and prolonged cytoplasmic bridge between GSC and cystoblast specify the fate of the future oocyte; however, this has not been demonstrated experimentally (reviewed in Huynh, 2006).

### An Intrinsic Timer Likely Regulates Fusome-Orchestrated Mitotic Divisions of the Cystoblast/Cystocytes

Cystoblast/cyst divisions are inherently different than those of the GSC (Figure 3). The GSC doubles in size prior to cytokinesis (King, 1970). In contrast, individual cystocytes in a 16-cell cyst are one-fifth of the volume of the cystoblast, indicating that the cystoblast exhibits reductive divisions. Where GSCs are severed from cystoblasts during cytokinesis, cystoblasts/cystocytes do not complete abscission and remain interconnected throughout the life of the cyst (Ong and Tan, 2010). GSCs also cycle continuously, while cytoblasts divide exactly four times. These comparisons have led researchers to speculate that cystoblasts autonomously limit the number of divisions through a molecular counting mechanism (King, 1970; McKearin, 1997; Huynh, 2006).

As in the GSC, the cell cycle machinery clearly underlies cystoblast/cyst divisions and is a prime candidate for a counting mechanism. Cyclin/Cdk protein levels and activity are very high in early cyst divisions, but decrease as cysts approach the terminal mitotic division (Figure 3) (Lilly et al., 2000; Hinnant et al., 2017). Cyclin expression then resumes in meiotic 16-cell cysts as nurse cells initiate endocycling (Reed and Orr-Weaver, 1997; Lilly et al., 2000). CycA becomes sub-cellularly localized at the fusome at the onset of cystoblast division, likely aiding in synchronous divisions (Lilly et al., 2000). Loss of CycE/Cdk2, CycB, or CycA block cystoblast division (Wang and Lin, 2005; Ables and Drummond-Barbosa, 2013; Flora et al., 2018), whereas overexpression of CycE (as occurs in *encore*, *dacapo*, and *Cullin 1* mutants, which fail to degrade CycE), CycA (as occurs in *effete* mutants, which fail to degrade CycA), or CycB results in a fifth cystoblast division (Lilly et al., 2000; Doronkin et al., 2003; Ohlmeyer and Schüpbach, 2003; Narbonne-Reveau and Lilly, 2009). Moreover, the accumulation of CycB is actively suppressed in differentiated nurse cells, ensuring mitotic exit (Reed and Orr-Weaver, 1997).

A key feature of the eukaryotic cell cycle is that Cyclin protein levels are rapidly degraded to promote entry into the succeeding cell cycle phase. This is accomplished by the interconnected activity of ubiquitin ligase complexes (reviewed in Thurlings and De Bruin, 2016; Werner et al., 2017; Yamano, 2019). The Cullin 4-containing Cullin-RING E3 ubiquitin ligase (CRL4) targets the transcription factor E2f1, signaling exit from S-phase. The anaphase promoting complex/cyclosome (APC/C) signals the metaphase/anaphase transition and mitotic exit by targeting CycB and CycA. In both cases, ubiquitination of key substrates targets those proteins for destruction by the 26S proteasome. During cystoblast/cyst divisions, activity of CRL4 and APC/C are very high at the 4- and 8-cell cyst stages, delaying cell cycle timing (Figure 3) (Hinnant et al., 2017). Intriguingly, this activity coincides well with the expression of the differentiation factor Bam (McKearin and Ohlstein, 1995). Indeed, a recent study demonstrated that Bam functions together with Ovarian Tumor (Otu) as a deubiquitinase, stabilizing CycA expression in dividing cysts (Ji et al., 2017). Over-expression of Bam forces a fifth mitotic division, resulting in cysts with 32-cells; conversely, failure to degrade CycA blocks cyst division (Chen et al., 2009; Ji et al., 2017).

In other systems, Cdk1 activates the APC/C by phosphorylating core APC/C subunits, including Cdc20 (reviewed in Yamano, 2019). This initiates a negative feedback loop wherein the APC/C regulates its own inactivation by targeting the M-phase cyclins for destruction. Thus, one possible model is that Bam/Otu creates an intrinsic timer in dividing cysts by stabilizing CycA in cystoblasts and 2-cell cysts, promoting subsequent APC/C activity and destruction of CycA in 4- and 8-cell cysts, triggering the terminal mitotic division. The Bam/Otu timer could be reinforced by other mechanisms that either activate the APC/C, such as Gcn5-induced acetylation (Liu et al., 2017), or regulate CycA levels, such as reduction of *CycA* mRNA by the CCR4-NOT deadenylase complex (Morris et al., 2005; Fu et al., 2015; Sgromo et al., 2018) and the translational repressor Bruno (Sugimura and Lilly, 2006).

## Specifying the Oocyte: Selection of the Oocyte Within the Cyst and Onset of Meiosis

The chromosomal events of meiosis are clearly integral to the formation of functional oocytes. Elegant cytologic studies demonstrated that critical meiotic events occur concurrently with mitotic divisions within a developing cyst (Figure 3) (Carpenter, 1975). Yet despite decades of study, molecular coordination between mitotic division and meiotic induction is not well-understood. Recent reviews have thoroughly described the intricate details of *Drosophila* female meiosis, and we refer readers there for comprehensive reading (Rubin et al., 2016; Hughes et al., 2018). Here, we highlight a few key features of this process relevant to the coordination of cell cycle and cell fate, and in particular, the role of the fusome during these events.

In meiosis, diploid cells undergo two rounds of chromosome segregation. Homologous chromosomes must pair, initiate and resolve crossovers, and then move to the right place in the cell to facilitate proper segregation to daughter cells (Gerton and Hawley, 2005). Meiosis is also an important catalyst for genetic variation. Thus, oocytes must be able to support homologous recombination (Hughes et al., 2018). In *Drosophila*, homologous chromosome pairing is excluded in primordial germ cells and does not commence in adult germ cells until the premeiotic stages of oogenesis, after the mature ovary is formed (Tanneti et al., 2011; Christophorou et al., 2013, 2015; Joyce et al., 2013). GSCs are the first to undergo chromosome pairing and as cyst divisions progress, chromosome pairing increases, with 8-cell cysts containing the most paired marks. Once pairing is established and double-strand DNA breaks (DSBs) are introduced, meiotic recombination occurs. Early cysts exiting the premeiotic stage and entering early pachytene begin to form a tri-partite proteinaceous structure called the synaptonemal complex between paired chromosomes (reviewed in Hughes et al., 2018). The synaptonemal complex consists of cohesins, cohesin-related complexes, as well as the central region proteins C(3)G, Corolla, and Corona. Assembly and disassembly of the synaptonemal complex is regulated by the E3 ubiquitin ligase encoded by *Seven in absentia* (Hughes et al., 2019). Since many of the mechanisms of meiosis are conserved but modified from mitosis, it is perhaps not surprising that cohesins are essential for both mitotic division and meiotic induction in developing oocytes (Khetani and Bickel, 2007; Gyuricza et al., 2016). Synaptonemal complex proteins can be visualized as early as the 4-cell cyst stage, first restricted to the two original cells created by the first cystoblast division, and then dispersing through at least four total cystocytes as cyst division progresses (Figure 4C) (Tanneti et al., 2011). It is worth noting that the pro-oocytes are likely also the first cystocytes produced, exhibiting the most intracellular bridges to other cystocytes, the most ring canals, and the fusome material of the cyst cells (Figure 3) (reviewed in Huynh, 2006).

As described above, Bam, Otu, and CycA play important roles in timing the mitotic divisions. Bam/Otu may also link cyst division and meiotic induction via the interaction of Bam with mei-P26 (Li et al., 2013; Ji et al., 2017). In addition to severely decreased rates of meiotic recombination and increased chromosome non-disjunction, ovaries from *mei-P26* mutants have two predominant phenotypes: a tumorous ovary phenotype (resembling *bam* mutants) and accumulation of egg chambers with 32-cell cyst (resembling a fifth cyst division) (Page et al., 2000). The fusome likely spatially coordinates the intrinsic timer in dividing cysts, as Bam and CycA co-localize at the fusome (McKearin and Ohlstein, 1995; Lilly et al., 2000). Indeed, many of the cell cycle mutants described above also fail to properly localize oocyte-specific factors, highlighting the importance of cell cycle state in fate specification (Huynh and St Johnston, 2004).

As cysts enter mid-pachytene, marks of DSBs such as γ-H2Av occur at the sites of SCs (Mehrotra and McKim, 2006). Subsequent break repair indicates completion of meiotic recombination. The timely and successful processes of DSB formation and repair are critical for oocyte identity and the production of high-quality oocytes (reviewed in Hughes et al., 2018). The molecular details of DSB repair in *Drosophila* oogenesis have provided mechanistic information regarding cellular conditions in which repair does not properly occur, such as cancer. The meiotic checkpoint that senses and repairs DSBs involves many evolutionarily conserved genes, including *grapes* (*Chk1* homolog), *mei-41* (*ATR* homolog), *loki*/*mnk* (*Chk2* homolog), *telomere fusions* (*ATM* homolog), *spindle-B* (*XRCC3* homolog), *spindle-A* (*Rad51C* homolog), *okra* (*Rad54L* homolog), and *Brca2* (Ghabrial et al., 1998; Ghabrial and Schüpbach, 1999; Abdu et al., 2002; Klovstad et al., 2008; Joyce et al., 2011). Generally, mutation of these genes results in a delay in oocyte specification and a reduction in Gurken (Grk) translation, thereby resulting in dorsal-ventral patterning defects (González-Reyes et al., 1997; Ghabrial et al., 1998; Ghabrial and Schüpbach, 1999). Intriguingly, however, delayed and/or aberrant DNA repair is not sufficient to result in a loss of oocyte identity, as is seen when SC formation is disrupted. This suggests that the oocyte is able to “overlook” defects in establishing the meiotic prophase I arrest, but not misorganization of cytoplasmic components.

## Oocyte Determination: Polarization and Accumulation of Oocyte-Specific Factors

In *Drosophila*, both the anterior-posterior and dorso-ventral axes of the future embryo are set up during oogenesis and the formation of these axes have been studied extensively (Schüpbach and Wieschaus, 1991; González-Reyes et al., 1995; Roth et al., 1995) and reviewed recently (McLaughlin and Bratu, 2015; Merkle et al., in press). Although much of this patterning occurs during the later stages of oogenesis, the establishment of polarity within the newly formed 16-cell cyst is necessary for proper organization of the microtubule cytoskeleton as well as specification and maintenance of oocyte fate. One of the first signs of polarity within the germline cyst occurs when the selected oocyte translocates to the posterior end of the newly-formed egg chamber through upregulation of *E*-cadherin and is anchored to a subset of follicle cells at the posterior terminus of the egg chamber called the posterior follicle cells (Godt and Tepass, 1998). Once the oocyte is established in the posterior of the cyst, additional polarization activities help traffic organelles, mRNA, and protein to the oocyte, establishing oocyte polarity and identity.

The fusome continues to feature prominently in dividing cysts, as it is essential for establishing a polarized microtubule array that aids in oocyte determination. Several microtubule motor and other interacting proteins have been implicated in both fusome formation and oocyte fate (McGrail and Hays, 1997; Liu et al., 1999; Wilson, 1999; Grieder et al., 2000; Máthé et al., 2003; Röper and Brown, 2004; Wehr et al., 2006). Mutation of the genes encoding these molecules, including Dynein heavy chain (Dhc), Lissencephaly-1 (Lis1), Orbit/Mast, Short stop (Shot), and Deadlock, result in aberrant oocyte specification and/or maintenance. Furthermore, the centrioles, mitochondria, and other organelles that form the Balbiani body, a conserved aggregate of organelles and oocyte-specific proteins and RNAs, are also trafficked between the cystocytes along the fusome and are important for establishing polarity of the cyst and oocyte (Grieder et al., 2000; Bolívar et al., 2001; Cox and Spradling, 2003). Finally, a few polarity-specific proteins, most notably Par-1, are associated with the fusome and may assist in or direct proper cyst and oocyte polarity establishment (Huynh et al., 2001a; Lighthouse et al., 2008). These factors and processes will be discussed further for their roles in cyst divisions and oocyte identity.

### Establishment of a Microtubule Organizing Center in the Cyst

A polarized microtubule network is essential for oocyte differentiation, mediating the transport of mRNAs and proteins through the actin-rich ring canals from the nurse cells to the presumptive oocyte (Theurkauf et al., 1993; Theurkauf, 1994a). For example, feeding female *Drosophila* with microtubule-depolymerizing drugs such as colchicine disrupts the accumulation of oocyte-specific factors in the pro-oocytes and results in an egg chamber with 16 nurse cells and no oocyte (Koch and Spitzer, 1983; Theurkauf et al., 1993). Moreover, germ cells harboring mutations in components of the dynein-dynactin complex, including *Dynein heavy chain 64C* (*Dhc64C*), *Lissencephaly-1* (*Lis-1*), and *Dynamitin* (also known as *Dynactin 2* and *DCTN2-p50*), give rise to 16 nurse cells and no oocyte (McGrail and Hays, 1997; Liu et al., 1999; Januschke et al., 2002). *Dhc64C* and *Lis-1* mutant ovaries contain abnormal fusomes, fail to migrate centrosomes to the oocyte, and exhibit oocyte specification defects, providing additional connections between the microtubule cytoskeleton, fusome biogenesis, and oocyte fate (McGrail and Hays, 1997; Liu et al., 1999; Swan et al., 1999; Bolívar et al., 2001).

As discussed above, microtubules and microtubule-associated proteins associate with the fusome in the early regions of the germarium, indicating that the fusome is critical for establishing the polarity of the microtubule network (Grieder et al., 2000). The fusome may serve as a central conduit for centrosome migration, a necessary first step in establishing a microtubule organizing center (MTOC) in the oocyte (Bolívar et al., 2001); reviewed in Megraw and Kaufman (2000). Interestingly, migration of centrosomes into the oocyte occurs normally after colchicine treatment, suggesting centrosomal migration is either microtubule-independent or only occurs along stable, pre-existing microtubules (Bolívar et al., 2001). Once the MTOC is established, it nucleates new microtubules that pass through the ring canals and into adjacent cystocytes, connecting the 16 cells of the cyst (Theurkauf et al., 1993). The fusome also coordinates the plane of cystocyte divisions by anchoring mitotic spindles. Indeed, spindles in *Dhc64C* mutant egg chambers fail to associate with the fusome, resulting in oocyte specification defects (McGrail and Hays, 1997; Bolívar et al., 2001). This may suggest that the fusome helps establish an asymmetry required for early oocyte identity.

### Establishing Polarity in the Cyst and Oocyte

In general, proteins responsible for establishing and maintaining cell polarity are typically associated with the cell cortex and localize asymmetrically to define different subcellular domains. The *Drosophila* homologs of the Par proteins, which include Par-1, Par-6, Par-3/Bazooka (Baz), and atypical Protein Kinase C (aPKC), have been shown to be key players in the generation of asymmetry and polarization within the early germline. Indeed, germline clonal analyses of *par-1* mutants have revealed that oocyte-specific RNAs and proteins initially localize properly to a single cell; however, oocyte identity is lost as this differential localization is disrupted in region 3 of the germarium (Cox et al., 2001; Huynh et al., 2001b). Interestingly, Par-1 seems to have a unique function in cyst polarity, as mislocalization or loss of the other polarity proteins does not result in cyst polarity defects. Instead, these mutants exhibit a failure to establish polarity within the oocyte once it is specified. In the germarium, Par-1 localizes to the fusome and ring canals within the cyst (Cox et al., 2001). After the disappearance of the fusome, Par-1 is restricted to the posterior cortex of the oocyte during oocyte specification, whereas the other polarity proteins, Par-6, Baz, Cdc42 and aPKC, localize to the anterolateral cortex of the selected oocyte (Vaccari and Ephrussi, 2002; Goldstein and Macara, 2007; Leibfried et al., 2013). Mutation of *par-1* disrupts microtubule organization and MTOC formation, thereby, preventing proper translocation of oocyte-specific RNAs and proteins from the anterior to the posterior of the oocyte (Shulman et al., 2000; Cox et al., 2001; Huynh et al., 2001b). This results in the oocyte reverting to the nurse cell fate, producing an egg chamber with 16 nurse cells and no oocyte, and ultimately arrests around stage 5 or 6 of oogenesis (Cox et al., 2001; Huynh et al., 2001b). Cdc42, a Rho-like GTPase, is an upstream regulator of Par protein localization and oocyte polarity maintenance (Leibfried et al., 2013). Mutants of *cdc42* and other polarity proteins such as *baz* and *par-6* exhibit a similar phenotype to *par-1* mutants, highlighting the importance of polarity on oocyte differentiation (Huynh et al., 2001a; Cox et al., 2002; Leibfried et al., 2013).

### Accumulation of Oocyte-Specific RNA Binding Proteins

Oocyte-specific factors such as *Bicaudal D* (*BicD*) and *oskar* (*osk*) mRNAs and Oo18 RNA-binding protein (Orb), BicD, Egalitarian (Egl), and fs(2)Cup (Cup) proteins preferentially accumulate in the oocyte by region 2b of the germarium (Figure 4D) (Wharton and Struhl, 1989; Ephrussi et al., 1991; Suter and Steward, 1991; Lantz and Schedl, 1994; Keyes and Spradling, 1997; Mach and Lehmann, 1997). During early stages of oogenesis, *orb* mRNA is localized at the posterior cortex of the oocyte. After repolarization of the microtubule network, *orb* mRNA concentrates along the anterior-lateral margin of the oocyte (Lantz and Schedl, 1994). In an *orb* null allele, the last mitotic division to form a 16-cell cyst does not occur, resulting in cyst degeneration. In less severe loss-of-function *orb* alleles, a 16-cell cyst forms, however, the egg chambers contain only nurse cells and no oocyte. Once the oocyte identity is specified, Orb protein accumulates at the poster cortex with its target mRNA *osk*. Disruption of the microtubule cytoskeleton has been shown to result in mislocalization of oocyte-specific factors including Orb, BicD, and Egl (Koch and Spitzer, 1983; Vaccari and Ephrussi, 2002).

Orb is a member of the conserved cytoplasmic polyadenylation element binding protein (CPEB) family and functions as a translational activator by promoting polyadenylation of localized mRNAs, although Orb can also act as a translational repressor in other contexts (Lantz and Schedl, 1994; Richter, 2007). In the oocyte, Orb regulates translation of oocyte-specific transcripts, including those important for axis formation, such as *osk* and *nanos* (Christerson and McKearin, 1994; Lantz and Schedl, 1994; Chang et al., 1999, 2001; Tan et al., 2001; Castagnetti and Ephrussi, 2003). Orb, which positively autoregulates its own translation, also functions in the assembly of the pole plasm at the posterior of the developing embryo (Ephrussi et al., 1991; Wang et al., 1994; Tan et al., 2001; Costa et al., 2005; Wong and Schedl, 2011). Given the many stages of oogenesis during which *orb* is required, it has been difficult to untangle the precise mechanism(s) by which Orb controls oocyte identity. Recent work, however, shows that the 3′UTR of *orb* mRNA is required in an autoregulatory mechanism for timely oocyte fate identity (Barr et al., 2019). When the 3′UTR of *orb* is removed or disrupted, mRNA accumulation and protein translation are aberrant, resulting in a failure to specify the oocyte. Proper localization of *orb* mRNA by the mRNA transport proteins BicD and Egl in the early dividing cyst is therefore required for oocyte fate. Given the wide array of mRNAs that Orb may translationally regulate, it will be interesting to elucidate which mRNAs encode for factors required for the molecular control of oocyte differentiation.

Cup, another translational control protein that interacts with Orb, is also important for oocyte identity and egg chamber polarity. During oogenesis, Cup associates with initiation factor eIF4E and Bruno to repress precocious translation of *osk* mRNA (Nakamura et al., 2004). In *cup* mutant egg chambers, *osk* mRNA and protein levels are reduced and mislocalized, thereby disrupting the formation of early anterior-posterior patterning events (Nakamura et al., 2004; Broyer et al., 2017). This ultimately results in egg chamber degeneration in mid-oogenesis. While *cup* mRNA is found in all germ cells during early oogenesis, Cup protein is localized to the selected oocyte of the 16-cell cyst in region 2a of the germarium (Keyes and Spradling, 1997). Bruno also accumulates in the oocyte by region 2b of the germarium (Webster et al., 1997). Despite these localization patterns, oocyte specification occurs properly in *cup* and *bruno* mutant ovaries, suggesting that Cup and Bruno are not oocyte determinants *per se* (Keyes and Spradling, 1997; Webster et al., 1997; Broyer et al., 2017). For similar reasons, *osk* is likely not an oocyte determinant either. *osk* translation is repressed until later stages of oogenesis, suggesting that Osk protein is not required for oocyte fate (Kim-Ha et al., 1995; Markussen et al., 1995). Non-coding roles for *osk* RNA in oogenesis have also been reported (Jenny et al., 2006; Kanke et al., 2015). Notably, *osk* RNA null alleles arrest in oogenesis much earlier than the stage at which *osk* is translated and exhibit karyosome defects (Rongo et al., 1995; Jenny et al., 2006). Nevertheless, *osk* RNA null mutant egg chambers properly specify oocytes, eliminating *osk* as an oocyte determinant (Jenny et al., 2006; Kanke et al., 2015).

BicD and Egl are two additional oocyte-specific cytoplasmic factors necessary for oocyte identity and RNA transport. These proteins form a complex with the minus end-directed MT motor dynein and loss of either results in egg chambers with 16 nurse cells and no oocyte (Suter et al., 1989; Schüpbach and Wieschaus, 1991). In *BicD* and *egl* null alleles, the fusome properly forms, but no asymmetry of cytoplasmic markers occurs within the cyst (Suter and Steward, 1991; Ran et al., 1994; Mach and Lehmann, 1997; de Cuevas and Spradling, 1998; Huynh and Johnston, 2000). Surprisingly, however, mutation of *BicD* and *egl* have opposing defects to result in this phenotype, suggesting that they have distinct functions. *BicD* mutants fail to localize other oocyte-specific proteins and RNAs to a single cystocyte (for example, *osk*, *orb*, and *fs(1)K10*), resulting in all 16 cystocytes becoming polyploid nurse cells (Suter and Steward, 1991; Ran et al., 1994). In *egl* mutants, however, all nuclei initially enter meiosis and accumulate oocyte-specific proteins and RNAs before reverting back to a nurse cell fate (Carpenter, 1994; Mach and Lehmann, 1997; Huynh and Johnston, 2000). Furthermore, they exhibit opposite defects in synaptonemal complex formation (Huynh and Johnston, 2000). BicD promotes synaptonemal complex formation in cystocytes that enter meiosis, while Egl represses complex formation in cystocytes that will become nurse cells. Strong *orb* mutants are similar to *egl* mutants in SC formation, suggesting that Orb protein may also be involved in this repression.

Interestingly, several oocyte-specific translational control proteins, including Cup, Bruno, and Orb associate with the BicD/Egl/dynein machinery to regulate RNA transport and/or translation during oogenesis (Nakamura et al., 2004; Clouse et al., 2008). BicD, Egl, and Dynein light chain (Dlc) form a complex that binds oocyte-specific mRNAs and proteins, such as Orb protein and *osk* mRNA, and transports them to the oocyte (Mach and Lehmann, 1997; Navarro et al., 2004; reviewed in: Vazquez-Pianzola and Suter, 2012). Egl interacts directly with Dlc, which transports the entire complex toward the minus ends of microtubules (Mach and Lehmann, 1997; Bullock and Ish-Horowicz, 2001; Navarro et al., 2004; Claußen and Suter, 2005; Dienstbier et al., 2009). BicD and Egl, however, are not associated with fusome and mutants do not exhibit defects in centrosomal migration, suggesting their functions are limited to transport in established cysts (de Cuevas and Spradling, 1998; Bolívar et al., 2001).

## Maintaining Oocyte Identity and Establishing the Maternally-Inherited Genome

Consistent with the developmental definition of “specification,” oocyte specification is reversible; therefore, once the oocyte has been specified, the identity must be properly maintained. If maintenance does not occur, the oocyte can exit meiosis, enter the endocycle, and take on a nurse cell-like fate. Mutant analyses of genes encoding conserved polarity and cytoskeletal proteins reveal the necessity of establishing and maintaining proper polarity within both the oocyte and the cyst in oocyte fate.

### Cytoskeletal Events

As discussed above, cytoskeletal machinery orchestrates critical events in *Drosophila* oogenesis, including cell division, the transport of molecules required for oocyte fate, and the formation of the future embryonic axes. Importantly, one of the first events required for maintenance of oocyte identity is the formation of a Balbiani body in the selected oocyte. The Balbiani body is a continuation of the formation of a MTOC in region 2a of the germarium, in which oocyte-specific RNAs and proteins migrate to the specified oocyte, along with the centrioles from the other 15 cystocytes. In early region 3, a Balbiani body made of mitochondria, Golgi vesicles, centrosomes, and oocyte-specific proteins and mRNAs forms at the anterior of the oocyte (Grieder et al., 2000; Cox and Spradling, 2003; reviewed in: Megraw and Kaufman, 2000). As the cyst rounds in late region 3/stage 1, the Balbiani body moves posteriorly to form a tight crescent at the posterior cortex of the oocyte. The movement of the Balbiani body posteriorly is the first sign of anterior-posterior polarity in the oocyte (Cox and Spradling, 2003).

Several microtubule-associated factors are also involved in maintenance of the oocyte identity. Orbit/Mast (also called Chromosome bows; Chb), the *Drosophila* homolog of CLASP, is a microtubule-associated protein that has been shown to interact with the mitotic spindle of the dividing cytocytes and the fusome (Máthé et al., 2003). Once egg chambers bud off from the germarium, Orbit/Mast protein is first concentrated in the oocyte cytoplasm, and then translocates into the oocyte nucleus around stage 6 of oogenesis. *orbit* mutant germaria show defects in stem cell maintenance, cystocyte divisions, fusome growth, ring canal establishment, and microtubule/MTOC organization. Many, if not all, of these defects are likely due to disruption of Orbit/Mast in cytoskeletal organization and establishing asymmetric spindle orientation during mitosis (Inoue et al., 2000; Lemos et al., 2000).

Like the microtubule network, the actin cytoskeleton is also essential to maintain oocyte identity. In fact, mutation of some actin-associated proteins results in microtubule cytoskeletal defects, suggesting they work together to coordinate processes of oogenesis. For instance, mutation of the actin nucleators *cappuccino* (*capu*) and *spire* (*spir*) results in subcortical MT bundles during oogenesis (Theurkauf, 1994b; Emmons et al., 1995; Wellington et al., 1999; Dahlgaard et al., 2007). One explanation for this interaction is that Capu and Spir help to organize cortical actin, which is required for Par-1 localization at the posterior of the oocyte (Doerflinger et al., 2006; Rosales-Nieves et al., 2006). Par-1 is an intriguing molecule in oocyte identity, as it associates with the fusome early in oogenesis before accumulating at the posterior cortex of the selected oocyte. Its localization, spatially and temporally, and its associations with diverse molecules suggest it may play different roles in oocyte identity.

### Chromosomal and Epigenetic Events

Mutant analyses show that when meiotic prophase I arrest is not maintained in oogenesis, oocyte fate identity is lost, resulting in entry to the endocycle. These studies have provided much-needed insight regarding the events that control the meiotic I arrest in *Drosophila* oogenesis. One important example is *missing oocytes* (*mio*), which was uncovered in a forward genetic screen for genes involved in oocyte differentiation. In *mio* mutant egg chambers, the oocyte initially enters meiosis, but fails to maintain the meiotic arrest, leading to a loss of oocyte identity (Iida and Lilly, 2004). Mutant oocytes instead enter the endocycle and develop as polyploid nurse cells. *mio* encodes a protein that accumulates in the pro-oocyte nuclei in early prophase of meiosis I and is then restricted to the selected oocyte. Mio genetically and physically interacts with Seh1 (Nucleoporin 44A), a conserved component of the nuclear pore complex (NPC) (Senger et al., 2011; Wei et al., 2014). Similar to *mio* mutants, *seh1* mutant egg chambers fail to specify an oocyte, leading to a cyst of 16 nurse cells (Senger et al., 2011). Studies show that Mio and Seh1 form the GTPase-activating proteins toward Rags 2 (GATOR2) complex, which acts to oppose the GATOR1 complex, thereby effectively positively regulating the target of the rapamycin complex 1 (TORC1) (Wei et al., 2014). How *mio* and *seh1* mechanistically control meiotic progression and oocyte identity, through the TOR pathway or otherwise, remains unclear.

Unsurprisingly, key cell cycle regulators have also been shown to be required for the prophase I arrested in the selected oocyte. One such factor is the p21^CIP^/p27^Kip1^/p57^Kip2^-like cyclin-dependent kinase inhibitor Dacapo (Dap), which accumulates in the oocyte nucleus to inhibit DNA replication and maintain the prophase I meiotic arrest (de Nooij et al., 2000; Hong et al., 2003). Dap inhibits Cyclin E (CycE) to maintain its meiotic state, as oscillations of CycE/Cdk2 activity are required for endoreplication in polyploid nurse cell nuclei. In *dap* mutant ovaries, the oocyte is initially specified, but soon enters the endocycle and develops into a nurse cell, thereby resulting in a loss of oocyte identity. Transcription of *dap* and *CycE* is suppressed by the Polycomb group (PcG) protein Polycomb repressive complex 2 (PRC2) during meiosis in *Drosophila* females (Iovino et al., 2013). PRC2, like other Polycomb-group proteins, silences gene expression by epigenetically modifying histone H3. Mutant clonal analyses of genes encoding two PRC2 components, Enhancer of zeste [E(z)] and Suppressor of zeste 12 [Su(z)12], revealed that PRC2 is required for oocyte fate maintenance. Similar to *dap* mutant egg chambers, mutation of *E(z)*, which encodes the catalytic methyltransferase subunit of PRC2, results in a loss of oocyte identity. While initial specification of the oocyte occurs, oocyte identity fails to be maintained and the cell enters the endocycle and becomes transcriptionally active, similar to nurse cells.

Transcriptional activity ceases in the selected oocyte during the early stages of oogenesis and only shortly reactivates during stages 9–10 (King and Burnett, 1959; Navarro-Costa et al., 2016). Moreover, the chromatin of the oocyte condenses to form a karyosome in order to maintain the required level of chromatin compaction to halt gene expression. Remarkably, the mechanisms controlling chromatin remodeling and gene expression regulation during oogenesis in *Drosophila* have yet to be deeply investigated. The Histone H2A kinase, NHK-1, is one of the few proteins in this category to be characterized during meiosis, particularly during karyosome formation. In *nhk-1* mutant ovaries, oocytes do not form a karyosome and the DNA instead aggregates at the nuclear periphery. Additionally, while the synaptonemal complex remains intact in *nhk-1* mutant oocytes, proteins required for condensation such as SMC4 are displaced from the DNA. These data indicate that the synaptonemal complex fails to dissociate from the meiotic chromosomes, thereby preventing proper condensation and karyosome formation. Furthermore, several histone post-translational modifications do not occur properly in *nhk-1* mutant oocytes, including specific phosphorylation of Histone 2A (H2AT119ph), suggesting this modification may be required for karyosome formation. The identification of these and other histone marks underline the importance and careful regulation of epigenetic modifications during female meiosis in *Drosophila* (reviewed in Ivanovska and Orr-Weaver, 2006).

One chromatin-associated protein essential for nurse cells is Suppressor of Hairy-wing [Su(Hw)], a DNA-binding protein that regulates the chromatin insulator *gypsy*. It is unclear if Su(Hw)’s role in oogenesis is directly related to this activity, as other gypsy insulator proteins are not required for oogenesis (Baxley et al., 2011; Soshnev et al., 2013). Su(Hw) protein localizes to the chromatin of the follicle cells and 15 polyploid nurse cells, but is restricted from the oocyte nucleus (Baxley et al., 2011). *su(Hw)* mutant egg chambers arrest in mid-oogenesis and undergo apoptosis, likely due to lack of oocyte growth (Klug et al., 1968; Baxley et al., 2011). Furthermore, *su(Hw)* mutant ovaries exhibit reduced ring canals, which may obstruct proper transport of critical factors from the nurse cells to the oocyte (Hsu et al., 2015). Future studies of Su(Hw) and other chromatin modifiers, both in the nurse cells and in the oocyte, are needed to gain deeper insight into the distinct mechanisms that control meiosis and the endocycle in *Drosophila* oogenesis.

## Open Questions and Concluding Remarks

In this review, we draw attention to the interconnected molecular mechanisms guiding cell division and cell fate. These themes are pervasive in cell and developmental biology, spanning from stem cell function and organ development to disease states such as tissue degeneration and cancer. Understanding how cell signaling and differentiation are molecularly integrated with cell cycle machinery during development is thus a fundamental challenge for the field. The formation of oocytes in *Drosophila* provides a sophisticated model within which to study acquisition of cell fate in a multicellular organism. Spatial and temporal coordination of cell proliferation and cell fate is necessary to ensure production of the correct quantity and quality of cells and, ultimately, optimum fertility.

The discovery that homologous chromosome pairing occurs prior to meiotic entry suggests that although they are tightly linked processes, oocyte selection can be uncoupled from pre-meiotic events (see Rubin et al., 2016). How cyst divisions are coordinated with meiotic entry and the timing of oocyte selection is an important topic for further study. Given the overlapping events of cyst formation, it is especially intriguing that the oocyte is selected from a pool of cells that share a common cytoplasm. What happens during these divisions to establish asymmetry? One central player is the fusome, which is directly implicated in cell division, oocyte establishment, and polarity. It is tempting to draw parallels between the fusome and asymmetrically distributed determinants in other systems. Asymmetric localization of proteins has been well-documented (particularly in *Drosophila* neuroblasts and the early *C. elegans* embryo) (Lerit et al., 2013; Ishidate et al., 2014). RNA, organelles, and histones have also been recognized to be asymmetrically distributed in dividing cells (Ouellet and Barral, 2012; Noatynska et al., 2013; Smyth et al., 2015; Eritano et al., 2017; Kahney et al., 2017; Yamashita, 2018; Shlyakhtina et al., 2019). Given the ER-like characteristics of the fusome, one possibility is that a centrosome-based microtubule mechanism partitions the fusome in dividing cystoblasts/cysts, reinforcing the initial asymmetry established between the GSC and cystoblast prior to abscission. Provided that the fusome is a membranous hub that docks the Balbiani body and the mitotic spindle, asymmetries associated with these structures could be necessary to establish initial cyst polarity. The fusome may thus fulfill the requirements of both oocyte specification models posited by pioneers in the field, acting as an “oocyte determinant” and biasing one germ cell toward the “winning” oocyte fate. Future studies should address how the fusome is partitioned in the dividing cyst cells and whether early asymmetric fusome inheritance pre-determines the presumptive oocyte. Live imaging studies may be necessary to elucidate these relationships.

Future studies investigating the role of the fusome are also essential to enhance our understanding of other aspects of cyst division, polarity, and oocyte growth. For example, what is the role of the fusome in timing cyst divisions? The fusome might spatially concentrate ubiquitin ligases, timing cyst division with the assembly of the synaptonemal complex. But it is not clear whether the fusome functions merely as a platform for cell division and acquisition of cell fate or has more active roles in the coordination of these processes. Moreover, once mitotic divisions are complete and cyst polarity is properly established, it is clear that oocyte fate is not yet determined and can be reversed. Persistence of fusome material jeopardizes cyst survival; this indicates that fusome dismantling, whose mechanism has yet to be explored, must faithfully occur to maintain egg chamber integrity and oocyte fate. Defects in cyst integrity have been described in mutant alleles of several trafficking or membrane-associated molecules, including Rab11, Sec5, and Cdc42 (Murthy et al., 2004; Bogard et al., 2007; Leibfried et al., 2013). These defects may be due to a failure to coordinate cyst polarity with cell growth. Even after oocyte selection and disassembly of the fusome, oocyte polarity and identity must both be maintained and these processes are not mutually exclusive. The chromosomal changes that direct pre-meiotic stages to progress into meiosis may also be related to the fusome, albeit in a less direct manner that the mechanisms above. It will be especially important to investigate epigenetic marks and chromatin structure during transition stages; in particular, when pre-meiotic nuclei exit S-phase, initiate meiosis, and then either remain in or exit meiosis. Once oocyte selection is complete, how are meiotic exit and endocycle entry coordinated in the appropriate cell? The “losing” pro-oocyte and the “winning” oocyte must engage in different cell cycle programs simultaneously, but have a shared cytoplasm. Proper orchestration of these different programs likely involves both cytoplasmic and nuclear control.

Lastly, the role of extrinsic signals in modifying the rate and quality of oocyte production cannot be overlooked. Escort cells in the germarium clearly send signals to the underlying germ cells as they divide, and insulin and ecdysone signaling in the escort cells are critical for escort cell structure and function. Whether and how hormonal signaling impacts oocyte differentiation is yet unknown. Another source of extrinsic signaling comes from the overlying somatic follicle cells. As egg chambers grow and mature, signaling between the germline and the surrounding soma is critical for egg chamber patterning and survival (reviewed in McLaughlin and Bratu, 2015; Merkle et al., in press). Disruption of the signaling crosstalk between these cell populations results in infertility or embryonic defects. Finally, during aging, cell cycle regulation changes, particularly in the GSCs, result in a reduction in the number of GSCs and an increase in cyst cell death. Meiotic errors also increase with age, which may be a direct result of GSC aberrations in aging females. Additional studies focusing on oocyte fate regulation and its coordination with cell cycle control in *Drosophila* will yield exciting new avenues for future research. Further insights may also provide a path to better understand human oocyte biology and infertility.

## Author Contributions

TH researched, wrote the parts of the manuscript, and made the figures. EA and JM conceived, researched, wrote, and edited the manuscript.

## Conflict of Interest

The authors declare that the research was conducted in the absence of any commercial or financial relationships that could be construed as a potential conflict of interest.
